# Global Assessment of Megavoltage External Beam Radiation Therapy Facilities: Current Status and Projections for 2030-2050

**DOI:** 10.1016/j.adro.2026.102014

**Published:** 2026-02-18

**Authors:** Fatemeh Sadat Tabatabaei, Amirali Azimi, Romina Abyaneh, Felipe Couñago, Filippo Alongi, Nina N. Sanford, Reza Ghalehtaki

**Affiliations:** a Center for Immunology and Inflammatory Diseases, Division of Rheumatology, Allergy and Immunology, Massachusetts General Hospital, Harvard Medical School, Boston, Massachusetts; b Radiation Oncology Research Center, Cancer Research Institute, Tehran University of Medical Sciences, Tehran, Iran; c Department of Radiation Oncology, Hospital Universitario San Francisco de Asís, GenesisCare, Madrid, Spain; d Department of Radiation Oncology, Hospital Universitario Vithas La Milagrosa, GenesisCare, Madrid, Spain; e Advanced Radiation Oncology Department, IRCCS Ospedale Sacro Cuore Don Calabria, Negrar, Italy; f University of Brescia, Brescia, Italy; g Department of Radiation Oncology, University of Texas Southwestern Medical Center, Dallas, Texas; h Department of Radiation Oncology, Cancer Institute, IKHC, Tehran University of Medical Sciences, Tehran, Iran

## Abstract

**Purpose:**

This study evaluates the worldwide distribution of radiation therapy (RT) facilities, focusing on current accessibility and projected needs at multiple future time points (2025, 2030, 2035, 2040, 2045, and 2050).

**Methods and Materials:**

Data were obtained from the World Bank, GLOBOCAN, and the International Atomic Energy Agency Directory of Radiotherapy Centres. A comprehensive projection model was developed that incorporates: (1) sensitivity analysis of machine throughput (patients per machine per year), (2) reirradiation rates stratified by country income level, (3) both country-specific and cancer-specific RT utilization rates, and (4) adjustments for the contribution of brachytherapy facilities.

**Results:**

Globally, there were 18.7 million new patients with cancer in 2022, of whom an estimated 11.2 million required RT. Of 215 countries and territories listed by the World Bank, 35 reported no data or zero RT facilities in Directory of Radiotherapy Centre, corresponding to a need for 362 MV RT machines by 2045. For the 146 countries with available data, 16,941 MV RT machines were reported, yielding a global patient coverage of 68% and a deficit of 7944 machines in 2022. Only 5.8% of the operating equipment is younger than 5 years. Projections indicate that by 2045, 16.7 million patients will require RT, necessitating 20,255 MV additional therapy units.

**Conclusions:**

This study underscores the persistent challenges in achieving equitable RT accessibility globally. The identified gaps extend beyond economic classifications, emphasizing the necessity for targeted interventions and strategic resource allocation to address the growing demand for RT and ensure comprehensive cancer care worldwide.

## Introduction

The influence of cancer has grown in importance in the dynamic field of global health. About 18.7 million new cases (not including nonmelanoma skin cancer) and 9.6 million fatalities were reported worldwide in 2022. This is a worrying trend that affects all quintiles of the Socio-demographic Index, with the lower quintiles showing the largest percentage increases.^1^ Over the next 10 years, it is anticipated that low- and middle-income nations will account for more than 75% of cancer-related deaths.^2^

Cancer demands a multifaceted approach to effective management. Among the various treatment modalities, radiation therapy (RT) stands as a cornerstone in cancer treatment: more than half of patients with cancer will receive RT during the course of illness, either as RT alone, or more frequently, in combination with other treatments, including surgery and/or systemic treatments.^3^^,^^4^ Thus, the accessibility and distribution of RT facilities worldwide present a critical issue, shaping the quality of care and outcomes. Despite its pivotal role, due to its dependence on cutting-edge, expensive technology, the global landscape of RT infrastructure remains marked by disparities, with significant gaps in equipment distribution and accessibility.^5^ This inequity primarily affects low-income and lower-middle-income countries.^6^^,^^7^ Understanding these disparities is crucial for devising strategic interventions to improve access to RT and global cancer care.^8^

Recent efforts, including a landmark analysis published in 2024, have provided robust estimates of global RT demand and workforce requirements in 2050.^9^ Although these studies have advanced the field by offering large-scale, population-based projections, they often rely on fixed assumptions regarding machine capacity, reirradiation, and RT utilization rates.^6^^,^^10^^,^^11^ Yet these parameters are not uniform across health systems and remain subject to considerable uncertainty, particularly when considering technological advances, variations in treatment practice, and the contribution of modalities such as brachytherapy. A comprehensive global assessment that explicitly examines how sensitive projected needs are to these underlying assumptions is still lacking.^12^^,^^13^ Addressing this gap is critical to ensure that planning for RT infrastructure captures both the scale of the challenge and the uncertainty that shapes future demand.

The main goal of this study is to map the current global distribution of megavoltage (MV) external beam RT machines in relation to cancer incidence, income level, and available infrastructure. In addition to providing a 2022 baseline, we project future RT needs at several time points (2030, 2035, 2040, 2045, and 2050). This approach allows health planners and policymakers to align short-, mid-, and long-term strategies. We also test how assumptions about machine capacity, reirradiation, utilization, and the role of brachytherapy affect the results, so that our estimates reflect a realistic range of possible future scenarios.

## Methods and Materials

### Study design

This study constitutes a comprehensive overview of existing data sources pertaining to RT facilities, cancer epidemiology, and the income levels of countries. The authors did not collect any primary data; instead, all information used in this study is sourced from publicly available data on the websites of relevant international agencies.

### Data sources

The incidence data for “all cancers excluding nonmelanoma cancers” and 36 specific cancer sites in each country were acquired from the GLOBOCAN, an initiative by the International Agency for Research on Cancer. The current cancer incidence statistics are based on the latest report in year 2022.^14^ Projected incidence rates for the years 2025, 2030, 2035, 2040, 2045, and 2050 were extracted from the Cancer Tomorrow section of the GLOBOCAN to estimate the anticipated requirements.^15^

The number and age of MV machines, along with data on brachytherapy and proton/light ion therapy units, were obtained from the International Atomic Energy Agency’s (IAEA) Directory of Radiotherapy Centres (DIRAC).^16^ Machine counts and ages (MV, brachytherapy, and proton/light ion) were taken from IAEA DIRAC. DIRAC is updated at different times by country; the latest available country-level entries used in this study range from 2018 to 2025 (Supplementary material [spread sheet]).

The list of countries, along with their gross national income per capita and corresponding income group classifications, was compiled from the World Bank.^17^ Income groups follow the Atlas method and the most recent classification (FY26, based on 2024 gross national income per capita) used thresholds of: low-income countries (LICs) ≤ US$1135; lower-middle-income countries (LMICs) US$1136-4495; upper-middle-income countries (UMICs) US$4496-13,935; and high-income countries (HICs) ≥ US$13,935. All data were accessed from the mentioned databases before August 1, 2025.

### Computation of RT capacity requirements

In their 2024 publication, Abu Awwad et al^6^ updated the evidence-based approach to RT utilization rates (RUR) for each cancer type, building on earlier studies that had primarily reported RUR values from high-income countries.^18^, ^19^, ^20^ Their analysis adjusted these rates for middle- and low-income settings and provided estimates for 24 common cancer sites, with the remainder (excluding nonmelanoma skin cancers) grouped as “others.” The detailed RURs are presented in the Supplementary material.

For each country, the incidence of each cancer was multiplied by the corresponding site- and income-specific RUR to estimate the number of patients requiring RT. RURs were recalculated for each year in our projection, reflecting variation by cancer type and country, which resulted in different overall RURs across countries and years. In accordance with European Society for Radiotherapy and Oncology - QUAntification of Radiation Therapy Infrastructure and Staffing Needs (ESTRO-QUARTS) and IAEA guidelines, 25% of RT cases were assumed to require reirradiation.^18^^,^^21^ However, recent evidence from Denmark suggests that reirradiation rates may be lower in some high-income settings.^22^ To account for this, we applied sensitivity analyses using income-specific ranges: 15% to 20% for high-income countries and 20% to 25% for middle- and low-income countries. The total number of patients requiring RT in each country and year was therefore calculated as: cancer incidence (excluding nonmelanoma skin cancer) × country- and year-specific RUR × reirradiation factor. An example of the indices calculated in this study is provided in Table 1.

**Table 1:** Examples of calculations for Iran

Index	Example (Iran)
New cancer cases requiring radiation therapy in 2022	(Bladder incidence × 53%) + (brain incidence × 80%) + (breast incidence × 77%) + … + (vulva incidence × 58%) + (other incidence × 53%) = 61,884
Radiation therapy utilization rate (RUR)	(New cancer cases requiring radiation therapy in 2022) ÷ all cancer cases (excl. nonmelanoma skin cancer) × 100 = (76,973 ÷ 133,121) × 100 = 46.3%
Total cancer cases requiring radiation therapy in 2022	(New cancer cases requiring radiation therapy in 2022) + (reirradiation cases) = 61,884 + (0.25 × 61,884) = 77,355
Radiation therapy capacity in each country	(Number of megavoltage machine supply) × 450 = 123 × 450 = 55,000
Radiation therapy patient coverage	(Radiation therapy capacity) ÷ (total cancer cases requiring radiation therapy) × 100 = 55,000 ÷ 77,355 × 100 = 72.0%
Optimal required megavoltage machine	(Total cancer cases requiring radiation therapy) ÷ 450 = 171
Megavoltage machine gap	(Optimal required megavoltage machine) – (MV machine supply) = 171 – 123 = 48
MV machine gap in 2045 to supplied MV machine in 2022	(MV machine gap in 2045) ÷ (MV machine supply in 2022) = 231 ÷ 123 = 1.87

*Abbreviation:* MV = megavoltage.

To determine the optimal number of MV machines required in the projected years and assess patient coverage in 2022, the IAEA guidelines were followed.^21^ Treatment capacity was calculated assuming 450 patients treated per MV machine per year, in accordance with IAEA guidelines, and the coverage percentage of patients was obtained by dividing this result by the estimated number of patients needing RT that year. For future projections, the required number of MV machines was estimated by dividing the projected number of patients by 450, and the gap in MV machines was calculated by subtracting the current number of machines from this requirement. While the main text presents estimations for 2045, calculations for earlier and later years (2030, 2035, 2040, and 2050) were also performed and are provided in the Supplementary spreadsheet. Sensitivity analyses were conducted using alternative machine capacities of 400 and 500 patients per MV unit to account for potential overutilization or underutilization of equipment. We used the online tool Mapchart.net to create geographic visualizations for the percentage of patient coverage in 2022.

In addition to MV machines, we conducted a separate analysis of brachytherapy capacity, which is detailed in the Supplementary Material. For this analysis, we assumed a capacity of 300 patients per high-dose-rate brachytherapy unit per year.^23^ Patient coverage and machine deficit estimates for 2022, as well as projected deficits for 2045, were adjusted accordingly to account for the contribution of brachytherapy to overall RT capacity.

To contextualize future infrastructure requirements, we calculated an additional descriptive indicator by dividing the projected MV machine deficit in 2045 by the number of MV machines currently in operation in each country. This ratio was used to reflect the relative scale of future expansion required compared with existing national RT capacity, based on historical investment levels. Importantly, this indicator is intended to facilitate cross-country comparison of proportional capacity gaps rather than to serve as a prescriptive planning metric.

## Results

### Data availability

The World Bank's list comprised 215 countries. For 30 countries (territories) listed by the World Bank, no data were available in either GLOBOCAN or DIRAC databases. These countries collectively accounted for a population of 11.1 million. Only Micronesia falls under the LMIC classification; the other 29 countries were all UMIC or HIC (Table 2).

**Table 2:** Countries (territories) listed on World Bank without available data on GLOBOCAN and DIRAC

No.	Country (territory)	GNI per capita	Income group	Population
1	American Samoa	-	High income	46,770
2	Andorra	48,870	High income	81,940
3	Antigua and Barbuda	21,380	High income	93,770
4	Cayman Islands	61,780	High income	74,460
5	Channel Islands	-	High income	168,126
6	Hong Kong SAR, China	57,100	High income	7,524,100
7	Curacao	20,990	High income	200,000
8	Dominica	10,220	Upper middle income	66,210
9	Faroe Islands	72,200	High income	54,720
10	Greenland	-	High income	56,840
11	Grenada	10,550	Upper middle income	117,207
12	Isle of Man	78,440	High income	84,160
13	Kiribati	3620	Lower middle income	134,518
14	Kosovo	7180	Upper middle income	1,500,000
15	Liechtenstein	-	High income	39,327
16	Marshall Islands	8380	Upper middle income	37,550
17	Micronesia, Fed. Sts.	4250	Lower middle income	113,160
18	Nauru	21,260	High income	11,950
19	Northern Mariana Islands	-	High income	44,280
20	Palau	14,070	Upper middle income	17,700
21	San Marino	53,930	High income	33,980
22	Seychelles	17,460	High income	121,354
23	Sint Maarten (Dutch part)	36,890	High income	43,350
24	St. Kitts and Nevis	22,310	High income	46,840
25	St. Martin (French part)	-	-	26,130
26	St. Vincent and the Grenadines	11,020	Upper middle income	100,616
27	Tonga	5520	Upper middle income	104,175
28	Turks and Caicos Islands	34,690	High income	46,540
29	Tuvalu	8770	Upper middle income	9600
30	Virgin Islands (U.S.)	-	High income	104,377
	Total	-	-	11,103,750

*Abbreviation:* DIRAC = Directory of Radiotherapy Centres.

Of the remaining 185 countries, 4 countries (territories) had data available on DIRAC but were devoid of information in GLOBOCAN. All 4 were HIC (Aruba, Bermuda, Macao SAR, Monaco), with a combined population of 0.9 million and collectively possessed a total of 6 MV RT machines.

Among the remaining 181 countries, 35 reported no available data on DIRAC or indicated zero RT facilities in the DIRAC database. These countries spanned all income groups and had an incidence of all cancer cases (excluding nonmelanoma skin cancer) totaling 123,382, with a deficit of 175 MV machines in 2022. These countries will require a total of 362 MV RT machines by 2045 (Table 3).

**Table 3:** Countries (territories) listed on World Bank with available data on GLOBOCAN but without available data on DIRAC

No.	Country (territory)	All cancer cases (excl. nonmelanoma skin cancer)	RUR (%)	MV machine gap in 2022	MV machine gap in 2045	Income group
1	Afghanistan	23,750	50.1	–33	–72	Low income
2	Belize	375	54.5	–1	–1	Upper middle income
3	Benin	7365	50.8	–10	–23	Lower middle income
4	Bhutan	630	46.4	–1	–2	Lower middle income
5	Burundi	7873	55.4	–12	–30	Low income
6	Cape Verde	423	56.3	–1	–2	Lower middle income
7	Central African Republic	2656	52.2	–4	–8	Low income
8	Chad	10,141	46.9	–13	–29	Low income
9	Comoros	599	60.7	–1	–2	Lower middle income
10	Congo, Republic of	2717	51.4	–4	–9	Lower middle income
11	Djibouti	787	51.0	–1	–2	Lower middle income
12	Equatorial Guinea	910	53.3	–1	–4	Upper middle income
13	Eritrea	2403	50.0	–3	–7	Low income
14	Eswatini	1073	62.2	–2	–4	Lower middle income
15	Fiji	1593	50.9	–2	–3	Upper middle income
16	French Polynesia	877	52.3	–1	–3	High income
17	Gaza Strip and West Bank	4978	49.2	–7	–16	High income
18	Guam	412	51.5	–1	–1	Lower middle income
19	Guinea	8558	52.4	–12	–27	Low income
20	Guinea-Bissau	1141	51.8	–2	–4	High income
21	Haiti	13,753	47.9	–18	–29	Lower middle income
22	Lesotho	1969	61.0	–3	–5	Lower middle income
23	Liberia	3796	52.8	–6	–11	Low income
24	Maldives	471	53.6	–1	–2	Low income
25	New Caledonia	1123	52.2	–2	–3	Upper middle income
26	Saint Lucia	448	54.7	–1	–1	High income
27	Samoa	400	52.2	–1	–1	Upper middle income
28	Sao Tome and Principe	143	50.1	0	0	Lower middle income
29	Sierra Leone	1915	50.8	–3	–6	Lower middle income
30	Solomon Islands	681	50.7	–1	–2	Low income
31	Somalia	10,423	51.7	–15	–33	Lower middle income
32	South Sudan	6737	52.8	–10	–17	Low income
33	The Republic of the Gambia	1192	48.0	–2	–4	Low income
34	Timor-Leste	820	52.0	–1	–2	Low income
35	Vanuatu	250	46.3	0	–1	Lower middle income
	Total	123,382	51.6	–175	–362	

*Abbreviations:* DIRAC = Directory of Radiotherapy Centres; MV = megavoltage; RUR = radiation therapy utilization rates.

Finally, there were 146 countries listed in the World Bank with available data both on DIRAC and GLOBOCAN.

### RT capacity and patient access in 2022

Globally, there were 18.6 million patients newly diagnosed with cancer with an RUR of 49.7%, resulting in an overall estimate of 11.2 million patients needing RT in 2022. The latest reported number of MV RT machines worldwide was 16,941, resulting in a global patient coverage of 68% assuming 450 patients treated per MV machine per year. Overall, there was a deficit of 7944 MV RT machines in 2022.

Thirty-one countries exhibited patient coverage equal to or exceeding 100% in 2022. Along with the 35 countries with no reported RT machine on DIRAC, implying 0% patient coverage, an additional 14 countries demonstrated patient coverage below 10%.

The patient coverage among HICs ranged from 62% to 273% with an overall patient coverage of 103.2%, indicating a surplus of 319 MV machines relative to the estimated need in 2022. The patient coverage among UMICs was 50.0% (ranging from 14.9% to 186.6%) with a deficit of 5194 MV machines. In LMICs, the patient coverage was 34% (ranging from 3.7% to 116.8%), indicating a deficit of 2946 MV RT machines. In LICs, the patient coverage was 7.9%, with 10 countries below 10% coverage, and 13% was the highest coverage in this income level. Additionally, there was a deficit of 541 MV RT machines in 2022. A detailed breakdown of the patient coverage in 2022 for each country is provided in Table 4.

**Table 4:** Each country’s cancer cases requiring radiation therapy in 2022 and projected numbers for 2045, and gaps in megavoltage (MV) machine

	2022							2045				
Country	All cancer cases (excl. nonmelanoma skin cancer)	RUR (%)	Cancer cases requiring RT (new + reirradiation)	MV machine supply	RT patient coverage* (%)	MV machine gap	MV therapy machine with age <5 years (%)	All cancer cases (excl. nonmelanoma skin cancer)	RUR (%)	Cancer cases requiring RT (new + reirradiation)	MV machine gap	Gap to currently available MV machine
High-income countries												
Australia	151,529	47.2	85,800	224	117	33	0.0	230,121	46.9	129,571	–64	0.3
Austria	45,525	49.5	27,066	58	96	–2	1.8	57,913	48.9	33,974	–17	0.3
Bahamas	937	51.9	583	1	77	0	0.0	1788	50.5	1083	–1	1.4
Bahrain	1373	51.9	855	2	105	0	0.0	3533	51.8	2194	–3	1.4
Barbados	1104	49.9	661	1	68	0	0.0	1291	49.5	767	–1	0.7
Belgium	72,680	51.5	44,916	99	99	–1	1.0	92,333	51.1	56,566	–27	0.3
Brunei Darussalam	902	47.7	516	2	174	1	0.0	1843	46.8	1035	0	0.2
Canada	232,074	50.1	139,424	296	96	–14	2.4	336,725	49.8	201,336	–151	0.5
Chile	57,082	45.1	30,901	45	66	–24	33.3	101,113	45.0	54,553	–76	1.7
Costa Rica	11,817	44.1	6251	12	86	–2	25.0	21,036	44.2	11,167	–13	1.1
Croatia	27,512	47.7	15,747	28	80	–7	32.1	29,345	47.3	16,655	–9	0.3
Cyprus	5921	48.8	3470	10	130	2	5.0	9755	48.9	5722	–3	0.3
Czechia	61,107	48.0	35,229	67	86	–11	0.0	69,179	47.6	39,528	–21	0.3
Denmark	43,964	48.3	25,473	55	97	–2	10.9	54,320	47.9	31,246	–14	0.3
Estonia	7817	48.2	4518	10	100	0	50.0	9047	47.7	5179	–2	0.2
Finland	35,274	49.9	21,111	50	107	3	14.0	41,521	49.3	24,559	–5	0.1
France	435,114	50.5	263,484	570	97	–16	11.9	523,982	49.8	313,147	–126	0.2
Germany	529,955	50.0	318,027	566	80	–141	0.0	637,317	49.5	378,256	–275	0.5
Greece	63,176	50.2	38,036	58	69	–27	10.3	73,211	49.9	43,823	–39	0.7
Hungary	62,509	50.4	37,770	52	62	–32	23.1	70,042	50.1	42,143	–42	0.8
Iceland	1663	49.7	992	2	91	0	0.0	2602	49.1	1534	–1	0.7
Ireland	26,884	50.5	16,299	42	116	6	0.0	42,422	49.8	25,333	–14	0.3
Israel	30,234	49.1	17,822	37	93	–3	13.5	48,826	48.8	28,569	–26	0.7
Italy	407,240	48.5	237,153	542	103	15	15.2	485,984	48.0	280,105	–80	0.1
Japan	992,806	46.7	556,332	1069	86	–167	0.6	1,047,154	46.3	581,553	–223	0.2
Korea, Rep.	232,963	43.0	120,231	191	71	–76	2.4	349,173	43.3	181,430	–212	1.1
Kuwait	4327	49.0	2544	5	88	–1	0.0	16,534	47.2	9360	–16	3.2
Latvia	11,082	48.1	6403	15	105	1	33.3	10,958	47.9	6294	1	–0.1
Lithuania	16,119	49.1	9494	16	76	–5	18.8	16,582	48.6	9661	–5	0.3
Luxembourg	2983	52.1	1865	4	96	0	0.0	5204	51.9	3241	–3	0.8
Malta	2738	47.9	1575	3	86	–1	0.0	4187	47.2	2371	–2	0.8
Netherlands	116,224	49.8	69,410	154	100	0	3.9	143,048	49.2	84,466	–34	0.2
New Zealand	27,551	47.8	15,791	35	100	0	5.7	42,073	46.8	23,646	–18	0.5
Norway	37,449	47.8	21,487	43	90	–5	7.0	54,384	47.4	30,911	–26	0.6
Oman	3991	45.9	2200	7	143	2	71.4	8106	46.3	4502	–3	0.4
Panama	8110	47.0	4570	7	69	–3	0.0	14,942	46.9	8409	–12	1.7
Poland	202,037	51.0	123,537	172	63	–103	2.9	254,474	50.5	154,157	–171	1.0
Portugal	66,600	48.5	38,783	57	66	–29	3.5	77,221	47.8	44,250	–41	0.7
Puerto Rico (US)	13,173	49.4	7814	21	121	4	0.0	17,029	49.0	10,006	–1	0.1
Qatar	1719	46.0	949	4	190	2	50.0	3475	46.2	1926	0	0.1
Romania	100,471	49.9	60,102	110	82	–24	45.5	112,157	49.3	66,322	–37	0.3
Russian Federation	614,275	48.0	353,847	569	72	–217	15.1	698,808	47.6	399,470	–319	0.6
Saudi Arabia	27,768	43.1	14,363	45	141	13	11.1	70,666	42.8	36,325	–36	0.8
Singapore	24,801	47.8	14,225	28	89	–4	7.1	46,700	46.6	26,094	–30	1.1
Slovak Republic	29,273	48.6	17,059	24	63	–14	8.3	39,309	47.9	22,580	–26	1.1
Slovenia	13,719	48.7	8015	12	67	–6	8.3	17,166	47.7	9832	–10	0.8
Spain	264,528	49.5	157,074	262	75	–87	1.5	348,808	48.6	203,450	–190	0.7
Sweden	61,794	48.0	35,582	80	101	1	3.8	80,155	47.7	45,916	–22	0.3
Switzerland	48,153	50.1	28,974	85	132	21	1.2	67,684	49.8	40,443	–5	0.1
Trinidad and Tobago	3918	50.5	2372	7	133	2	28.6	6207	50.1	3729	–1	0.2
United Arab Emirates	5445	48.0	3137	19	273	12	22.7	15,611	48.0	8999	–1	0.1
United Kingdom	417,481	50.9	254,882	358	63	–208	7.5	543,254	50.4	328,401	–372	1.0
United States of America	1,832,550	50.4	1,107,601	3892	158	1431	4.3	2,442,122	50.2	1,470,226	625	–0.2
Uruguay	15,664	49.7	9346	22	106	1	13.6	20,016	49.5	11,887	–4	0.2
*Total*	7,513,105	49.0	4,421,669	10,145.0	103.2	319	6.3	9,518,456	48.7	5,557,902	–2206	0.2
Upper middle-income countries												
Albania	7702	53.9	5188	5	43	–7	0.00	10,294	53.6	6902	–10	2.1
Algeria	63,306	52.4	41,491	37	40	–55	0.00	118,003	51.5	76,015	–132	3.6
Argentina	129,689	50.6	82,018	134	74	–48	0.00	196,261	50.5	123,785	–141	1.1
Armenia	9397	48.7	5725	5	39	–8	60.00	11,449	47.6	6816	–10	2.0
Azerbaijan	18,176	50.8	11,541	10	39	–16	0.00	29,367	51.0	18,716	–32	3.2
Belarus	45,019	49.7	27,943	31	50	–31	12.90	52,472	49.4	32,387	–41	1.3
Bosnia-Herzegovina	13,765	51.0	8770	15	77	–4	40.00	14,969	50.4	9432	–6	0.4
Botswana	2222	59.3	1648	3	82	–1	66.67	4341	60.0	3256	–4	1.4
Brazil	591,660	52.1	385,384	374	44	–482	0.00	988,382	52.3	645,662	–1061	2.8
Bulgaria	31,485	48.6	19,145	43	101	0	20.45	30,978	48.1	18,627	2	0.0
China	4,775,419	47.7	2,849,553	2932	46	–3400	0.65	6,988,919	49.2	4,294,472	–6611	2.3
Colombia	114,573	48.8	69,932	95	61	–60	4.21	202,406	48.9	123,696	–180	1.9
Cuba	45,705	57.3	32,713	20	28	–53	0.00	58,891	56.8	41,825	–73	3.6
Dominican Republic	19,887	55.1	13,693	21	69	–9	0.00	33,839	54.9	23,207	–31	1.5
Ecuador	29,613	47.1	17,441	35	90	–4	28.57	56,862	47.3	33,603	–40	1.1
El Salvador	9600	48.5	5825	15	116	2	20.00	13,902	48.1	8367	–4	0.2
Gabon	1842	53.0	1221	2	74	–1	0.00	3885	53.8	2610	–4	1.9
Georgia	13,184	50.5	8324	18	97	0	0.00	13,088	50.2	8211	0	0.0
Guatemala	17,304	44.8	9694	8	37	–14	50.00	33,966	44.9	19,081	–34	4.3
Indonesia	400,820	51.7	258,826	86	15	–489	18.60	625,625	51.4	402,196	–808	9.4
Iran, Islamic Rep.	133,121	46.3	76,973	123	72	–48	0.00	279,801	45.5	159,264	–231	1.9
Iraq	36,982	50.8	23,476	54	104	2	14.81	87,424	51.4	56,190	–71	1.3
Jamaica	7429	55.6	5168	5	44	–6	0.00	9997	55.3	6908	–10	2.1
Kazakhstan	34,116	51.0	21,741	53	110	5	9.43	49,409	50.6	31,264	–16	0.3
Libya	8099	50.4	5099	9	79	–2	33.33	16,982	49.5	10,501	–14	1.6
Malaysia	51,133	50.3	32,176	71	99	–1	7.04	97,712	50.3	61,395	–65	0.9
Mauritius	2795	51.9	1813	5	124	1	40.00	3852	51.9	2500	–1	0.1
Mexico	198,533	47.3	117,349	207	79	–54	6.28	340,916	47.8	203,846	–246	1.2
Moldova	13,377	50.1	8372	4	21	–15	25.00	11,383	49.8	7086	–12	2.9
Mongolia	6651	30.7	2549	6	106	0	16.67	14,856	30.3	5622	–6	1.1
Montenegro	2510	55.5	1741	3	78	–1	33.33	3024	54.5	2060	–2	0.5
Namibia	3222	54.7	2202	2	41	–3	0.00	5896	55.3	4079	–7	3.5
North Macedonia	7235	53.6	4846	6	56	–5	33.33	9366	52.9	6196	–8	1.3
Paraguay	13,380	52.2	8728	11	57	–8	18.18	21,581	52.2	14,085	–20	1.8
Peru	69,765	46.7	40,688	65	72	–25	6.45	118,228	46.3	68,459	–87	1.3
Serbia	40,596	52.6	26,717	39	66	–20	20.51	36,681	52.3	23,972	–14	0.4
South Africa	100,897	56.3	71,049	102	65	–56	0.00	175,227	56.5	123,804	–173	1.7
Suriname	1103	52.6	726	3	186	1	0.00	1922	52.1	1251	0	–0.1
Thailand	180,337	46.7	105,289	151	65	–83	19.87	274,130	46.2	158,465	–201	1.3
Turkiye	231,784	50.6	146,478	298	92	–28	5.03	402,876	51.4	258,991	–278	0.9
Turkmenistan	6561	51.5	4220	7	75	–2	0.00	11,660	51.1	7447	–10	1.4
Ukraine	150,687	50.2	94,572	76	36	–134	28.95	155,951	50.0	97,424	–140	1.8
Venezuela	58,613	52.5	38,493	54	63	–32	9.26	94,309	52.5	61,865	–83	1.5
Total	7,699,294	48.8	4,696,537	5243	50	–5194	4.0	11,711,082	49.7	7,271,540	–10,916	2.1
Lower middle-income countries												
Angola	24,085	53.5	16,098	3	8	–33	0.00	57,369	54.4	39,003	–84	27.9
Bangladesh	166,093	58.6	121,644	37	14	–233	5.41	311,649	58.5	227,965	–470	12.7
Bolivia	16,811	49.2	10,340	16	70	–7	25.00	25,341	49.9	15,802	–19	1.2
Cambodia	19,516	44.0	10,726	3	13	–21	33.33	39,523	43.5	21,479	–45	14.9
Cameroon	19,260	56.5	13,592	2	7	–28	0.00	42,338	56.9	30,094	–65	32.4
Cote D'Ivoire	21,083	53.8	14,166	4	13	–27	0.00	44,798	54.3	30,394	–64	15.9
Egypt	148,639	43.7	81,164	124	69	–56	1.67	283,825	43.4	153,828	–218	1.8
Ghana	27,054	49.2	16,644	6	16	–31	0.00	55,511	49.9	34,613	–71	11.8
Honduras	10,663	48.5	6464	8	56	–6	12.50	21,116	49.3	13,001	–21	2.6
India	1401,721	56.2	983,955	806	37	–1381	6.01	2,435,314	56.4	1,716,747	–3009	3.7
Jordan	12,164	50.7	7702	20	117	3	15.00	27,201	51.1	17,364	–19	0.9
Kenya	44,065	55.9	30,806	17	25	–51	11.76	98,140	56.2	68,933	–136	8.0
Kyrgyz Rep.	7040	48.5	4268	1	11	–8	0.00	12,194	48.2	7348	–15	15.3
Lao P.D.R.	8993	43.7	4907	1	9	–10	0.00	17,448	43.5	9487	–20	20.1
Lebanon	12,690	52.2	8278	21	114	3	9.52	14,825	52.8	9783	–1	0.0
Mauritania	3202	51.7	2068	4	87	–1	0.00	6443	52.1	4193	–5	1.3
Morocco	63,110	55.4	43,735	49	50	–48	8.16	101,364	55.4	70,217	–107	2.2
Myanmar	76,043	51.3	48,761	22	20	–86	0.00	117,037	50.8	74,387	–143	6.5
Nepal	21,766	49.1	13,360	12	40	–18	25.00	37,126	49.5	22,977	–39	3.3
Nicaragua	8242	47.2	4866	5	46	–6	0.00	16,027	47.4	9504	–16	3.2
Nigeria	124,735	56.8	88,500	17	9	–180	17.65	253,882	56.9	180,728	–385	22.6
Pakistan	180,938	53.1	120,078	76	28	–191	14.06	329,463	53.6	220,556	–414	5.4
Papua New Guinea	11,491	52.2	7495	2	12	–15	0.00	24,547	52.1	16,000	–34	16.8
Philippines	187,806	49.1	115,312	80	31	–176	21.25	351,294	49.4	216,725	–402	5.0
Senegal	11,561	51.8	7483	4	24	–13	25.00	25,832	51.5	16,633	–33	8.2
Sri Lanka	32,829	52.9	21,710	29	60	–19	24.14	45,616	53.7	30,626	–39	1.3
Tajikistan	6366	46.1	3667	2	25	–6	0.00	12,910	45.0	7266	–14	7.1
Tanzania	44,070	59.5	32,785	10	14	–63	20.00	110,924	60.3	83,665	–176	17.6
Tunisia	20,095	53.5	13,435	32	107	2	21.88	33,963	52.9	22,475	–18	0.6
Uzbekistan	35,498	50.9	22,596	19	38	–31	15.79	60,894	50.5	38,448	–66	3.5
Viet Nam	179,123	46.5	104,142	70	30	–161	11.43	289,507	46.3	167,595	–302	4.3
Zambia	15,037	55.4	10,414	3	13	–20	0.00	38,083	56.8	27,033	–57	19.0
Zimbabwe	17,312	56.2	12,172	1	4	–26	0.00	37,484	56.6	26,514	–58	57.9
Total	2,979,101	53.8	2003,334	1506	34	–2946	9.13	5,378,988	54.0	3,631,382	–6564	4.4
Low-income countries												
Burkina Faso	14,308	43.7	7819	2	12	–15	100.00	32,373	44.5	18,010	–38	19.0
Congo, Dem. Rep.	52,049	54.0	35,138	1	1	–77	0.00	113,154	54.3	76,734	–170	169.5
Ethiopia	77,790	49.9	48,533	3	3	–105	33.33	170,684	50.6	107,877	–237	78.9
Guyana	1223	53.5	818	1	55	–1	0.00	1737	53.7	1166	–2	1.6
Korea, Dem. P. Rep.	61,212	48.6	37,193	3	4	–80	0.00	95,624	48.5	57,921	–126	41.9
Madagascar	20,693	56.3	14,569	3	9	–29	0.00	46,537	57.0	33,181	–71	23.6
Malawi	19,475	57.9	14,096	2	6	–29	50.00	42,761	58.9	31,508	–68	34.0
Mali	14,842	51.0	9470	2	10	–19	50.00	34,358	51.0	21,921	–47	23.4
Mozambique	25,741	53.0	17,056	1	3	–37	0.00	54,466	53.7	36,527	–80	80.2
Niger	11,263	42.8	6026	1	7	–12	0.00	27,290	42.8	14,601	–31	31.4
Rwanda	6966	51.3	4467	2	20	–8	0.00	14,621	51.9	9483	–19	9.5
Sudan	28,076	50.6	17,771	6	15	–33	0.00	57,395	50.7	36,379	–75	12.5
Syrian Arab Republic	21,505	51.9	13,962	13	42	–18	23.08	56,085	52.2	36,588	–68	5.3
Togo	5292	53.2	3519	1	13	–7	0.00	11,347	53.3	7562	–16	15.8
Uganda	35,539	54.6	24,263	5	9	–49	60.00	80,192	55.4	55,578	–119	23.7
Yemen, Rep.	16,260	49.0	9969	1	5	–21	0.00	37,437	49.1	22,988	–50	50.1
Total	412,234	51.4	264,671	47	8	–541	23.91	876,061	51.9	568,024	–1215	25.9
World	18,603,734	49.7	11,198,350	16,941	68	–7,944	5.80	27,484,587	50.2	16,737,987	–20,255	1.2

*Abbreviations:* RT = radiation therapy; RUR = radiation therapy utilization rates.

⁎ Assuming 450 patients treated per MV machine per year. Reirradiation rate was used as 0.2 for high-income countries and 0.25 for all other countries.

**Figure 1:**
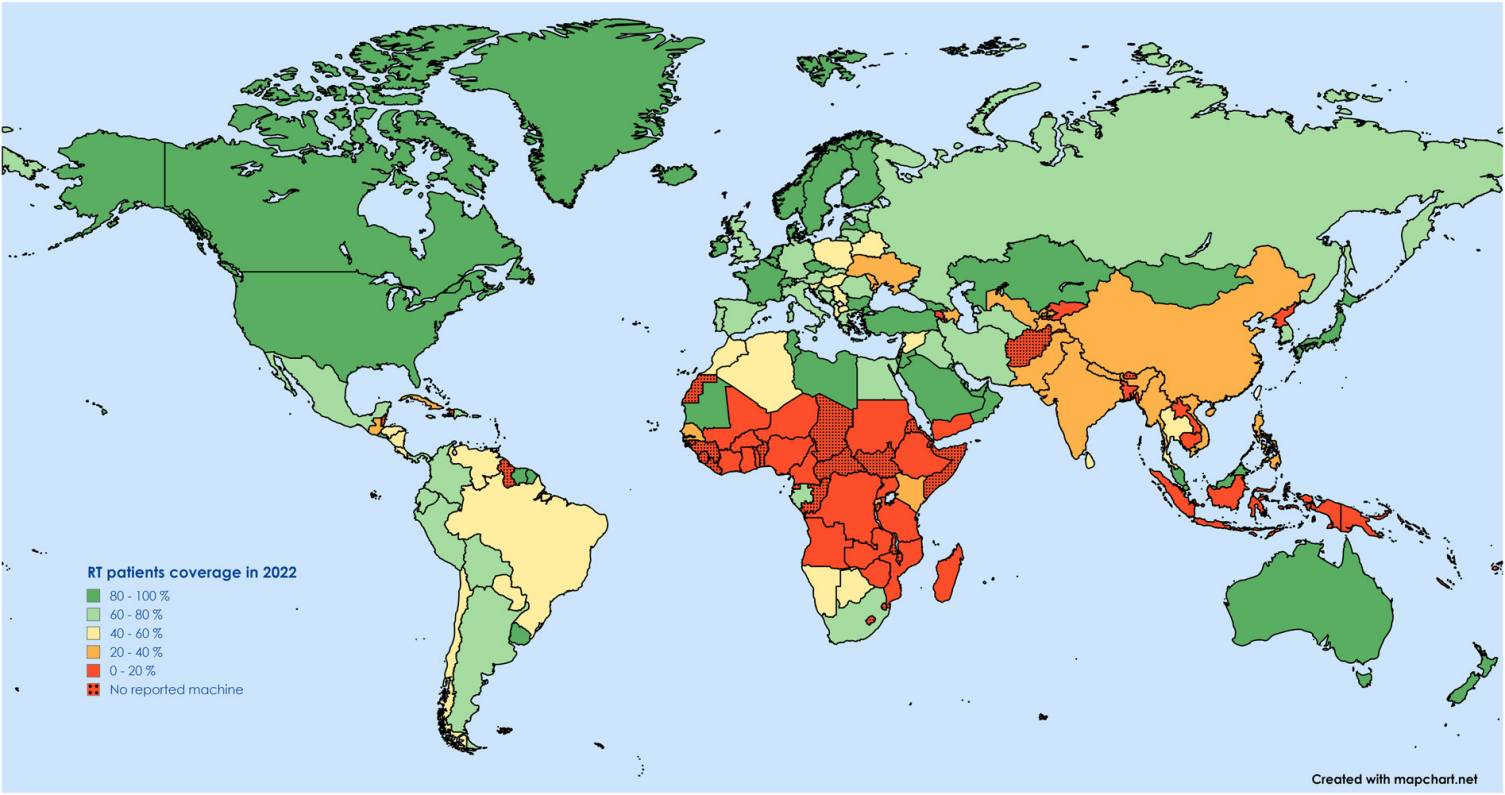
This map presents the geographic distribution of the percentage of radiation therapy (RT) patient coverage in each country in 2022.

The geographic distribution of patient RT coverage in 2022 is illustrated in Figure 1. While countries in Southern and Northern Africa demonstrated better patient coverage, the majority of Western, Eastern, and Central African countries exhibited patient coverage lower than 20%. In Asia, countries with coverage lower than 40% were more prevalent in Southeastern and Central Asia. Countries with patient coverage ranging from 40% to 60% were primarily situated in Eastern Europe, Central America, and various countries in Africa and Asia.

### Projected RT unit requirements for 2045

In 2045, it is projected that there will be approximately 27.5 million cancer cases globally (excluding nonmelanoma skin cancer), with an estimated 16.7 million patients requiring RT. It is anticipated that the addition of 20,255 MV RT units to the existing numbers will be required. However, this need is not evenly distributed. Seven countries currently have sufficient MV RT units for the projected needs in 2045. In contrast, as previously mentioned, there are 35 countries without reported RT units, resulting in a 100% deprivation of patients. Furthermore, LMICs and LICs are in need of an additional 6564 and 1215 MV machines by 2045, respectively, representing 38% of the total global requirement.

To facilitate the interpretation of projected infrastructure needs for 2045, we assessed the ratio of projected MV machine deficits to the number of currently available MV machines for each country. This indicator highlights the proportional magnitude of future capacity expansion required relative to existing infrastructure. Overall, the ratio of MV machine deficits to the currently available facilities in HICs, UMICs, LMICs, and LICs is 0.2, 2.1, 4.4, and 25.9, respectively. The details are shown in Table 4.

### MV therapy equipment age

Globally, data regarding the age of the equipment are not available for 2026 MV therapy units. Based on the available data, it can be observed that only 5.6% of the equipment are less than 5 years old. The units that are currently more than 5 years old will surpass 25 years in age by 2045. The percentage of equipment aged less than 5 years, based on the available data, is presented for each country in Table 4.

### Impact of brachytherapy on RT capacity

Incorporation of brachytherapy increased estimated RT patient coverage in 2022, primarily in settings where baseline coverage remained below 100% (Table 5). Globally, patient coverage rose from 68.0% before adjustment to 81.5% after adjustment for brachytherapy. In high-income countries, where coverage already exceeded 100%, brachytherapy did not meaningfully change access estimates, reflecting capacity saturation rather than unmet need. In contrast, upper-middle-income countries experienced a substantial increase in coverage (from 50.0% to 77.0%), while gains in lower-middle-income and low-income countries were modest, with coverage remaining below 30% and 10%, respectively. Detailed country-level results are provided in the Supplementary material.

**Table 5:** Patient coverage and machine deficit after adjustment for brachytherapy

Income level	Brachytherapy units	MV therapy (units)	Patient coverage 2022 (%) before adjustment for brachytherapy	Patient coverage 2022 (%) adjusted for brachytherapy	MV machine deficit 2022 before adjustment for brachytherapy	MV machine deficit 2022 adjusted for brachytherapy	MV machine deficit 2045 before adjustment for brachytherapy	MV machine deficit 2045 adjusted for brachytherapy
High-income countries	2057	10,145	103.2	114.0	+319	+2103	−2206	−322
Upper-middle-income countries	831	5243	50.0	77.0	−5194	−4223	−10,916	−9719
Lower-middle-income countries	457	1506	34.0	28.0	−2946	−2464	−6564	−5936
Low-income countries	6	47	8.0	10.0	−541	−513	−1215	−1161
World	3351	16,941	68.0	@#@	−7944	−5097	−20,255	−17,138

*Abbreviation:* MV = megavoltag.

### Further projections

In addition to the main analyses presented here, supplementary calculations are provided in an accompanying Excel file. These include projections for intermediate years 2030, 2035, 2040, and 2050 as well as sensitivity analyses incorporating alternative assumptions for reirradiation (15%-20% vs 20%-25%) and machine utilization (400, 450, or 500 patients per MV unit per year). This approach yielded 6 sensitivity matrices per projection year, the results of which are presented in the Supplementary material.

## Discussion

The results of the current study highlight a significant gap in the availability of optimal RT devices, affecting both developing and even some developed countries. The primary goal of this study is to bring attention to this critical situation among policymakers. Our findings revealed that, globally in 2022, only 68% of patients would have access to RT devices, with coverage ranging from 0% to 100% across different countries. This disparity underscores the unfavorable global situation and the urgent need for intervention in many countries. Previous studies have emphasized the necessity of providing additional equipment, particularly in lower and middle-income countries.^5^^,^^7^^,^^10^ However, our results revealed that the gap in RT requirements and availability is also substantial among some high-income countries. The current study underscores the reality that, despite previous efforts to enhance RT accessibility,^8^^,^^10^^,^^13^ a significant gap persists in the present situation.

Previous studies have assessed the availability of RT services, especially MV RT machines, in certain countries.^5^^,^^10^^,^^13^^,^^24^^,^^25^ However, predicting for 2030 may be too short-sighted for health policymakers, whereas projections for 2050 and beyond raise concerns about the uncertainties of technological changes in cancer treatment. In our study, we chose 2045 as the main long-term projection horizon. Importantly, we also provided estimates for all available timepoints in the Supplementary material. This approach enables policymakers to view projections across short-, medium-, and long-term horizons, making the results more actionable depending on their local needs and planning cycles.

Many studies focused on a limited number of countries within a single region, such as African or Asian countries, or only lower-middle-income countries.^5^^,^^6^^,^^12^ This restricts the ability to compare countries on a global scale comprehensively. Furthermore, health policymakers might compare their country's conditions with those of peer countries based on income and welfare categories and find it satisfactory, but the situation may still fall far short of global ideals. However, despite receiving similar titles like lower-middle-income, countries may still differ significantly in terms of RT coverage.

Some previous articles in their calculations have not included reirradiation patients,^6^^,^^12^ despite prior discussions about the workload they impose on RT infrastructure.^18^^,^^19^ In the current study, we have included reirradiation patients to achieve a more comprehensive view of coverage. Moreover, in a recent article,^6^ for their calculation of patient coverage, they considered 288 and 409 patients per MV machines for high-income countries and middle and low-income countries, respectively.^6^ While even a 1:450 ratio demonstrates large coverage gaps in most countries, we performed sensitivity analyses using 1:400 and 1:500 to account for potential underutilization or overutilization scenarios.^21^

Policies in developed countries commonly assume that access to RT is apparently sufficient. The emphasis in these countries is often placed on optimizing access within the national landscape.^26^^,^^27^ This approach aims to improve the overall accessibility of RT and aligns with the concerns of policymakers in these developed nations.^28^ However, in certain countries, the insufficient number of RT devices may result in the deprivation of some patients from receiving RT. In such cases, the fundamental policy within the treatment system should prioritize the addition of RT units. Subsequently, efforts can be directed toward ensuring the proper distribution of these devices based on geographic and population considerations.^24^

### Need for urgent action by 2045

This study also investigated the projected extent of deprivation of access to RT in 2045. It is anticipated that there is a global need to add about 20,000 new MV RT devices to the current number of devices globally. A total of 35 countries need to establish MV RT machines for the first time in their respective countries. As the majority of these countries belong to the low-income and lower-middle-income categories, there is a crucial need for support from global organizations, including the World Health Organization and IAEA, to expedite access in these nations. The ratio of projected MV machine deficits to currently available facilities provides important contextual insight into the scale and feasibility of future RT expansion. While absolute machine deficits convey the magnitude of unmet need, this ratio illustrates how disruptive those deficits may be relative to existing infrastructure. For example, a similar absolute shortfall may represent a manageable incremental expansion in settings with substantial installed capacity, but a fundamental system-level challenge in countries with minimal baseline infrastructure. Among the countries already equipped, 91 nations require adding a number of units exceeding their existing total. This situation implies that policymakers in these countries must allocate more budget and effort in the next 20 years to increase unit numbers than has been allocated since the introduction of RT in their country.

Equipment age is a critical aspect in evaluating RT facilities. While adding new units can help bridge the gap between supply and demand, the aging of existing equipment poses a challenge. Established guidelines suggest that no more than 17% of installed equipment should be over 10 years old.^29^ While these guidelines may seem stringent from a global perspective, our findings indicate that approximately 70% of current equipment will surpass this age threshold by 2035. Even in countries with minimal gaps between RT machine need and supply, aging equipment poses a significant threat to RT availability.

Furthermore, sophisticated techniques, including intensity modulated RT and volume modulated arc therapy, both usually integrated with image guided RT, are needed for treating several cancers like head and neck cancers and pelvic cancers, and these can be achieved with newer RT equipment.^7^ Additionally, advancements in systemic cancer treatments may result in an increased need for RT, re-RT, and palliative RT in the coming years.

While our study primarily addressed the availability of equipment, it is imperative to acknowledge the equally critical need for an increase in human resources, particularly RT staff, to operate the estimated number of RT machines. While previous studies have offered insights into the projected requirements for RT specialists and staff,^10^ there is a pressing need for updated assessments in this area. This need is particularly pronounced in countries that currently lack reported RT units, as the addition of RT equipment in the coming years will necessitate trained specialists to operate these newly established RT units.

Brachytherapy is an essential modality for the treatment of selected malignancies, most notably cervical cancer, and its inclusion provides important contextual information for RT capacity assessments. However, brachytherapy is inherently indication-specific and cannot substitute for external beam RT across the broad spectrum of cancers requiring RT.^30^ Our analysis shows that although brachytherapy modestly increases estimated patient coverage in some settings, it does not resolve the underlying structural deficit in MV RT capacity, particularly in low- and lower-middle-income countries. These findings reinforce that expansion of MV RT infrastructure is the primary prerequisite for achieving equitable global access, with brachytherapy functioning as a necessary but complementary component of comprehensive cancer care.

Several complementary strategies have been proposed to address disparities in RT access, ranging from cancer prevention and treatment optimization to infrastructure expansion and technological innovation. These approaches are summarized in panel 1, while the present discussion focuses on the central finding that expansion of MV RT capacity remains the primary requirement for achieving equitable global access.

### Panel 1: strategies and initiatives to address the global RT coverage gap

#### Cancer prevention and incidence reduction

One potential logical approach to mitigate the coverage gap is by reducing the need for RT. Cancer prevention programs have the capacity to alter the incidence composition of various cancer types within countries. Initiatives such as smoking cessation education can reduce the incidence of lung cancer.^31^ Similarly, in many developing countries, cervical cancer presents a high demand for RT and offers an effective prevention option.^32^^,^^33^ This reduction can lead to a decrease in the overall need for RT. Yet, in countries needing more RT infrastructure, cancer prevention programs might not fully compensate for the current gap due to delayed reductions in cancer cases. This underscores the urgent need to augment the number of RT equipment.^34^

#### Overutilization of existing RT infrastructure

In some countries with a small gap between the optimal and available number of devices, this gap is often compensated for by overworking the devices.^25^^,^^35^ Short-term increases in patient coverage can be achieved by extending device hours. However, concerns arise about device aging and increased workload, which could lead to higher chances of device failure and long-term retirement. Ultimately, this could reduce patient coverage in the long run. Therefore, the fundamental solution lies in increasing the number of devices.

#### Hypofractionated RT approaches

Another introduced modality is hypofractionated RT, achieved by increasing the dose of each fraction while decreasing the total number of fractions.^36^^,^^37^ This reduction in RT sessions may theoretically increase patient coverage with the available devices.^38^

#### Manufacturer pricing and market constraints

Manufacturing companies prioritize technological development, but can contribute to increasing global patient coverage by providing RT devices at more affordable prices, especially in lower-income countries. However, policymakers cannot rely solely on manufacturers to reduce prices, as these companies are prone to financial crises when working on the development of newly designed, expensive products. An example of such an imbalance occurred with ViewRay MRIdian in 2023,^39^ demonstrating that an exclusive emphasis on reducing prices may lead to a halt in technological development in RT treatment.

#### Global and regional capacity-building initiatives

Over the last decade, numerous initiatives have sought to address disparities in RT access. These include global efforts by the IAEA and World Health Organization, regional collaborations such as the African Radiation Oncology Network, and national programs that have expanded RT capacity through targeted funding and infrastructure development.^40^ Our findings highlight that, despite these advances, significant disparities remain, particularly in low- and middle-income countries. Beyond the number of machines, in-country barriers such as insufficient national funding, limited public insurance coverage, lack of quality assurance systems, and shortages in trained personnel continue to restrict equitable access.

#### Advancing the systemic cancer therapies

In parallel, advances in other cancer treatment modalities may influence future demand for RT. Immunotherapy and targeted therapies are expanding rapidly and, in some cancers, may change treatment paradigms.^41^ However, current evidence suggests that RT will remain essential, either as a primary modality or in combination with systemic therapies. Thus, while such treatments may modestly reduce the relative demand for RT, they cannot replace it in most settings.

#### Technological innovation and workflow efficiency

Technological innovation also offers opportunities to bridge the access gap. Developments in artificial intelligence, particularly in automated contouring and treatment planning, can improve workflow efficiency and throughput, partially offsetting workforce shortages.^42^ Similarly, novel machine designs such as compact LINACs (Linear Accelerators) and lower-cost cobalt-based systems may provide scalable solutions for LMICs.^43^ Continued integration of these technologies, alongside global and regional policy initiatives, will be critical for narrowing the gap between RT need and access by 2045.

### Limitations

The current study has some limitations. It relies on data from DIRAC, which, although periodically updated, is not uniformly current across countries. For 36 countries, the most recent updates were before 2021, which may affect the accuracy of present and projected RT access estimations. While it is recommended that each country calculate its RT coverage and access rates using the most recent national data, the figures derived from this study can serve as a practical baseline for needs assessment. Furthermore, the analysis assumes uniform distribution of RT equipment within countries, whereas significant regional disparities in access often exist. In addition, DIRAC focuses primarily on equipment numbers and does not comprehensively capture associated human resources, funding structures, or quality assurance programs, which are equally critical for the functional delivery of RT.

This assessment focuses on MV external beam RT machines, which constitute the majority of global RT capacity. Proton, light ion, and brachytherapy facilities are not included in the primary analysis because their global distribution remains limited and is concentrated predominantly in high-income countries. These factors should be considered when interpreting the projections presented in this work.

## Conclusion

In conclusion, this study emphasizes global disparities in RT infrastructure, revealing substantial gaps that threaten patient access. We projected a significant deficit in the number of RT machines, particularly in low-income and middle-income countries. The analysis underscores the urgency of addressing this shortage and emphasizes the importance of regular data updates. Immediate global collaboration is imperative to meet the demand for effective cancer care in the field of RT.

## Appendix Supplementary materials

Supplementary excel Image, application 1

## Footnotes

Supplementary material associated with this article can be found, in the online version, at doi:10.1016/j.adro.2026.102014.
